# Nonlinear Strength Reduction Method of Rock Mass in Slope Stability Evaluation

**DOI:** 10.3390/ma16072793

**Published:** 2023-03-31

**Authors:** Yifan Chen, Yizhou Chen, Hang Lin, Huihua Hu

**Affiliations:** 1 School of Resources and Safety Engineering, Central South University, Changsha 410083, China; 2 Hunan Provincial Communications Planning, Survey and Design Institute, Changsha 410200, China

**Keywords:** slope stability, nonlinear criterion, strength reduction method, cluster algorithm

## Abstract

As the strength parameters of rock mass degrade differently during slope instability, different factors should be considered in the strength reduction method. Previous nonlinear reduction methods were essentially implemented based on the Mohr–Coulomb criterion, which was reported not to reflect the nonlinear performance of rock mass. To address this deficiency, in this study, the Hoek–Brown criterion was combined with a nonlinear reduction technique for slope stability evaluation. Firstly, based on the classical definition of safety factors, the relationships that should be satisfied by each parameter of the critical slope were derived. The critical curve of the slope regarding the Hoek–Brown constant *m_b_* and the uniaxial compressive strength of rock mass *σ_cmass_* was then obtained. On the assumption that the slope parameter deterioration conforms to the shortest path theory, the reduction ratio of *σ_cmass_* to *m_b_* was determined. The more objective k-means algorithm was employed to automatically search the potential sliding surface, on which the slope safety factor was calculated as the ratio of sliding resistance to sliding force. Finally, the slopes in published literature were adopted for verification, and the calculated safety factors were compared with those by other methods, which showed better efficacy.

## 1. Introduction

**Figure 1:**
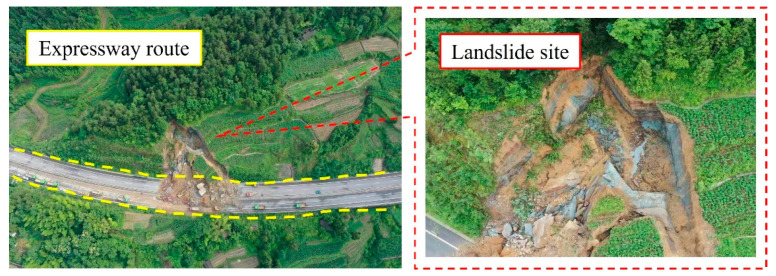
Historical landslide in expressway [10].

Landslides are one of the most dangerous geological disasters, causing heavy casualties and economic losses around the world [1,2,3,4]. In rock projects such as transportation, hydropower and mining, the original stable slopes are easily affected by the disturbance of engineering excavations [5,6,7,8], leading to more destructive disasters such as landslides and collapses [9], such as landslides occurred on the expressway [10] (Figure 1). The bearing capacity estimation has always been an important issue in the field of geotechnical engineering [11,12], which is also the basis for project design. In recent years, various numerical, analytical, as well as laboratory methods have been applied to evaluate the bearing capacity of slope rock mass [13,14,15], while slope stability analysis is an effective guarantee for the successful construction and safe operation of a project. In other words, the accurate assessment of slope stability is of great significance to reduce landslide risks, avoid geological disasters and reduce project investment [16,17,18].

Currently, both the limit equilibrium method and the strength reduction method are commonly used in slope stability calculations [19,20,21,22,23,24]. The former carries out static equilibrium analysis on rock mass slices based on the preassumed sliding surface to determine the minimum safety factor [25]. The strength reduction method, on the other hand, simultaneously reduces the cohesion *c* and the internal friction angle *φ* of the slope and regards the reduction factor corresponding to the instability as the safety factor of the slope [26,27,28,29], which is expressed in Equation (1).

(1) $$Fs=\frac{c^{initial}}{c^{critical}}=\frac{\tan\varphi^{initial}}{\tan\varphi^{critical}}$$

where *Fs* is the safety factor of slope, *c^critical^* and *φ^critical^* are respectively the cohesion and internal friction angle of critical slope, while *c^initial^* and *φ^initial^* are respectively the initial values of cohesion and internal friction angle of slope.

Compared with the limit equilibrium method, the strength reduction method has wider applicability and practicability in the stability analysis of projects such as mine slopes, highway subgrades, deep buried tunnels, and water conservancy dams, since it is simple in expression, convenient during calculation, and applicative to various geological conditions [30,31,32]. It was reported, however, that the traditional strength reduction method dismisses the different degradations of *c* and *φ*, and the reduction scheme is still controversial [33,34,35,36,37]. Tang and Zheng proposed the double safety factor method for slope stability analysis [38,39]. Pantelidis and Griffiths [40] also analyzed the influence of different reduction strategies on slope stability. Xue, Dang [41] derived the expression between reduction coefficients through linear attenuation assumption. Jiang, Wang [42] suggested that the reduction ratio of cohesion to internal friction should be 1.75 for homogeneous soil slopes. Yuan, Bai [43] fitted the reduction ratio of slope model with different slope angles and determined the specific reduction ratio by interpolation calculation. On the assumption that there are countless combinations of the reduction ratio of cohesion and internal friction angle, Isakov and Moryachkov [44] proposed the shortest reduction path theory to calculate the comprehensive safety factor of slope, which was then improved by Yuan, Li [45] based on critical *c*-tan *φ* curves. Fang, Chen [46] discovered a nonlinear instability criterion according to the concept of critical slope. These promote the rapid development of nonlinear strength reduction method for slope stability analysis, and all of them are implemented by linearly reducing the two Mohr–Coulomb parameters [47,48,49,50]. Meanwhile, rock mechanics gradually realized that the linear criterion cannot reflect the nonlinear failure characteristics of rock mass [51,52,53], in which case reduction methods based on the nonlinear Hoek–Brown strength criterion are constantly proposed [54].

For the description of the nonlinear performance of rock mass, Hoek and Brown proposed the Hoek–Brown strength criterion in 1980 based on abundant triaxial test results and engineering practice [55,56,57]. In 1992, Hoek et al. corrected the Hoek–Brown strength envelope of rock mass at low stress levels. Subsequently, the engineering application showed that it was too conservative in terms of rock mass with good quality. In this case, Hoek et al. [58,59] further improved the original results and proposed generalized Hoek–Brown strength criteria for rock mass of different quality, which is presented in Equation (2). In 2002, Hoek et al. [5,6,7,8] comprehensively discussed the Hoek–Brown parameter relations, and built a new determination method of parameters *m_b_*, *s* and *a* by introducing geological strength index *GSI* and disturbance factor *D* (see Equations (3)–(5)). At present, the Hoek–Brown strength criterion has been widely used in rock and rock mass mechanics analysis, rock slope stability analysis, rock tunnel design and other fields [60].

(2) $$\sigma_{1}-\sigma_{3}=\sigma_{ci}{\left(m_{b}\sigma_{3}/\sigma_{ci}+s\right)}^{a}$$

(3) $$m_{b}=m_{i}e^{\frac{GSI-100}{28-14D}}$$

(4) $$s=e^{\frac{GSI-100}{9-3D}}$$

(5) $$a=0.5+\left(e^{-GSI/15}-e^{-20/3}\right)/6$$

where *σ*_1_ and *σ*_3_ are, respectively, the major and minor principal stresses at rock mass failure; *m_b_*, *s*, and *a* are all the rock mass material constants; *m_i_* is a material constant, *GSI* is the geological strength index, and *D* is the disturbance factor ranging 0~1.

Current reduction methods based on the nonlinear Hoek–Brown criterion can be broadly classified into three categories: direct reduction, strength envelope lowering, and indirect reduction, which are summarized in Table 1. In direct reduction methods, Wu, Jin [61] believed that only the uniaxial compressive strength *σ_ci_* and material constant *m_i_* should be reduced when the nonlinear strength reduction method was used to calculate the safety factor of rock slope. Song, Yan [62] discussed seven direct reduction cases of stability calculation, and found that only by directly reducing the values of *σ_ci_* and *GSI* can the reasonable safety factor of a slope be obtained. Yang, Wang [63] selected Hoek–Brown parameters *σ_ci_* and *m_i_* as reducing objects to carry out a stability analysis of surrounding rock in a tunnel. For the second category, Hammah, Yacoub [64] lowered the envelope of the Hoek–Brown criterion to achieve global reduction. Lastly, Li, Merifield [65] adopted the equivalent Mohr–Coulomb parameters to achieve the indirect reduction of the Hoek–Brown criterion, and established the stability analysis chart of rock slope. Shen et al. [66,67] deduced the instantaneous equivalent Mohr–Coulomb parameters from the nonlinear Hoek–Brown criterion to implement safety factor calculation.

Each of these studies has contributed to the advancement of strength reduction methods based on the Hoek–Brown criterion. However, the above studies all use the same coefficients for the Hoek–Brown parameter reduction, which conflicts with the experimental evidence. To account for the degradation of the nonlinear strength parameter of the rock mass, this paper proposes a new nonlinear strength reduction method for slope stability evaluation. The selection of the Hoek–Brown parameter was achieved by establishing an expression for the safety factor of the Hoek–Brown parameter. The respective reduction coefficients were then derived based on the critical strength curve of the slope and the shortest path theory. Based on the horizontal displacement difference of the critical slope, the k-means algorithm was used to search for potential slip surfaces in order to calculate a more objective safety factor. As a result, a new nonlinear reduction method for Hoek–Brown material slopes was established and confirmed by comparison with other reduction methods.

## 2. Methods

### 2.1. Critical Strength Curve of Slope Satisfying Hoek–Brown Criterion

Slope stability is usually controlled by the geometrical parameters of the rock mass (such as slope height *H*, slope angle *θ*) and physical parameters (unit weight *γ*), as well as all parameters involved in the rock failure criterion. Taking the Hoek–Brown strength criterion as an example, the parameters include *σ_ci_*, *m_b_*, *s* and *a*. All of these should satisfy a certain functional relationship at the point of slope failure. According to the classical definition of the slope safety factor, it is computed by the sliding resistance and the sliding motion on the potential sliding surface. In another word, the slope safety factor is a function of the anti-sliding force and the sliding force on the potential sliding surface (see Equation (6)).

(6) $$Fs=f_{1}\left(\tau ,\tau_{g}\right)$$

where *τ* is the anti-sliding stress, *τ_g_* is the sliding stress, and *f*_1_ is an unknown function.

Generally, the sliding force on the potential sliding surface of the slope is provided by the weight of sliding mass (*Hγ*), thereby the slope safety factor *Fs* can be expressed as a function of slope height *H*, unit weight *γ* and slope angle *θ* (see Equation (7)).

(7) $$Fs=f_{2}\left(\tau ,\theta ,H\gamma \right)$$

The expression form of normal stress–shear stress (*σ_n_*-*τ*) on the failure surface of the Hoek–Brown strength criterion [68,69] is

(8) $$\sigma_{n}=\sigma_{3}+\frac{\sigma_{ci}{\left(m_{b}\frac{\sigma_{3}}{\sigma_{ci}}+s\right)}^{a}}{2+am_{b}{\left(m_{b}\frac{\sigma_{3}}{\sigma_{ci}}+s\right)}^{a-1}}$$

(9) $$\tau =\left(\sigma_{n}-\sigma_{3}\right)\sqrt{1+am_{b}{\left(m_{b}\frac{\sigma_{3}}{\sigma_{ci}}+s\right)}^{a-1}}$$

It can be seen from Equation (9), *τ* is closely related to Hoek–Brown strength parameter *σ_ci_*, *m_b_*, *s* and *a*, and normal stress *σ_n_*. Thus, *τ* can be expressed as Equation (10).

(10) $$\tau =f_{3}\left(\sigma_{ci},\sigma_{n},m_{b},a,s\right)$$

While the normal stress *σ_n_* on the potential sliding surface of the slope is also provided by the weight of sliding mass, which means

(11) $$\tau =f_{4}\left(\sigma_{ci},m_{b},a,s,\theta ,H\gamma \right)\;$$

By substituting Equation (11) into the slope safety factor expression Equation (7), the relationship between the safety factor *Fs* and slope parameters (see Equation (12)) can be obtained.

(12) $$Fs=f_{5}\left(\sigma_{ci},m_{b},a,s,\theta ,H\gamma \right)\;$$

According to the expression of the Hoek–Brown strength criterion (see Equation (2)), the uniaxial compressive strength of rock mass *σ_cmass_* can be estimated if *σ*_3_ = 0 (see Equation (13)). Therefore, the influences of parameters *a* and *s* on the slope stability are incorporated into that of parameter *σ_cmass_*.

(13) $$\sigma_{cmass}=\sigma_{ci}\cdot s^{a}$$

Combined with Equation (12), the slope safety factor *Fs* can be transformed into a function of the parameters *σ_cmass_* and *m_b_* as well as the physical and geometric parameters of the slope, as presented in Equation (14).

(14) $$Fs=f_{6}\left(\sigma_{cmass},m_{b},\theta ,H\gamma \right)\;$$

**Figure 2:**
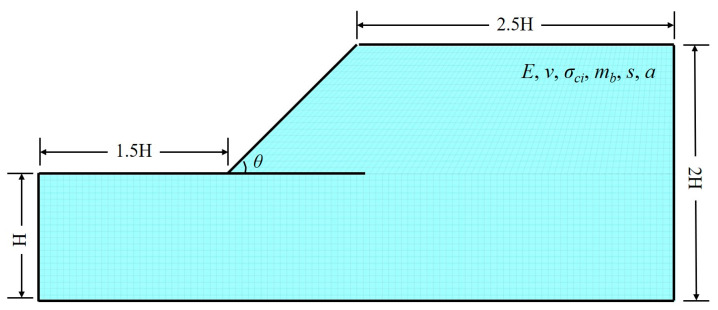
Geometric model of homogeneous slope.

For a given slope in critical state, all of the above parameters should satisfy the relationship of *Fs* = *f*_6_ (*σ_cmass_*, *m_b_*, *θ*, *Hγ*) = 1.0. Referring to the research results by Yuan et al. [45], there are numerous combinations of *c^critical^* and tan *φ^critical^* for a critical slope when it obeys the Mohr–Coulomb failure criterion [70,71,72], and the *c^critical^* and tan *φ^critical^* obtained by traditional strength reduction is only one potential possibility. In the same way, for a rock slope whose slope height *H*, weight *γ* and slope angle *β* have been determined, there are also numerous critical combinations of *σ_cmass_* and *m_b_*, which can also be plotted in the critical strength curve *σ_cmass_*-*m_b_*. The critical strength curve of the rock slope is mainly determined by numerical simulation of stability analysis on different slope models. Specific steps include:

(1) A series of homogeneous slopes with different slope angles *θ* and weight *γ* were established by FLAC^3D^, and the geometric model is shown in Figure 2. The slope height *H* = 20 m remained unchanged, the variation range in slope angle was set to 30°–75° with a gradient of 15°, and the variation range of unit weight was set to 20–26 kN/m^3^ with a gradient of 2 kN/m^3^.

(2) Referring to the built-in slope example in FLAC^3D^, other parameters are presented in Table 2.

(3) Different values of *m_b_* were assigned to slope models. In this study, it was linearly changed according to Equation (15).
  (15) $$m_{b}^{new}=m_{b}\times k$$
  where *m_b_^new^* is the value of the Hoek–Brown parameter *m_b_* for each slope model, *k* represents the variation coefficient, and *k =* 1, 5, 10, 15, 20, …, 100.

(4) The left and right boundaries were normally constrained, and model bottom was fixed in all directions. The initial stress field was generated by the gravity of the model and the default convergence condition was used as the indicator of slope failure. The value of *σ_ci_* was continuously reduced during the numerical simulation of stability analysis until the slope model reached the critical state for each slope model.
(5) The critical value of *σ_ci_* and the corresponding Hoek–Brown parameters of the slope were substituted to Equation (13) to calculate the specific value of the uniaxial compressive strength of rock mass *σ_cmass_*.
(6) Taking *m_b_* as the horizontal axis and *σ_cmass_* as the vertical axis, the critical strength curve of slope at any slope angle can be drawn. Finally, the general expression of slope critical strength curve at given slope angle can be obtained by nonlinear data fitting.

**Figure 3:**
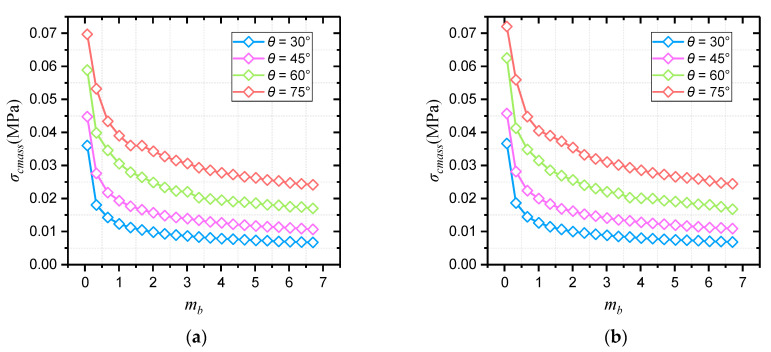

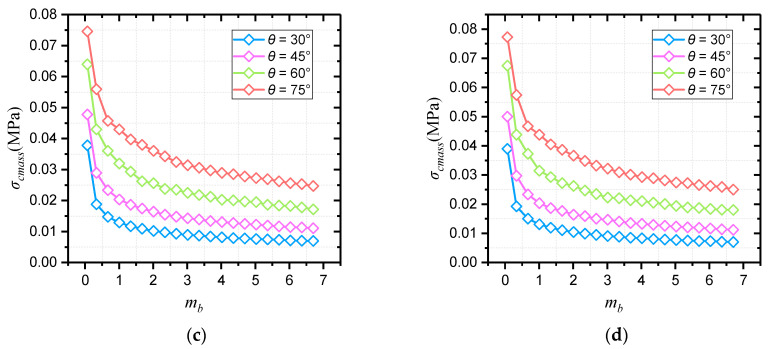
Critical Hoek–Brown strength curves of different slopes. (**a**) *γ* = 20 kN/m^3^; (**b**) *γ* = 22 kN/m^3^; (**c**) *γ* = 24 kN/m^3^; (**d**) *γ* = 26 kN/m^3^.

The critical strength curves of each slope under different unit weight and slope angle are shown in Figure 3.

For all the critical strength curves in Figure 3, the value of *σ_cmass_* drops with the increase in *m_b_*, and the dropping speed is decreasing. In principle, the slope stability is improved when the value of *m_b_* increases, and the required value of *σ_ci_* is lowered at slope critical state. As a result, the value of *σ_cmass_* drops according to Equation (13). Under the conditions of the same unit weight, greater strength is required to maintain slope stability at a greater slope angle. Therefore, the reduction factor of *σ_ci_* is less, and the critical value of *σ_cmass_* is greater. Reflected in Figure 3, the larger the slope angle, the higher the slope critical strength curve.

Since the slope critical strength curve is decreasing more and more slowly, it is assumed that all the curves in this study conform to the law of power function variation (see Equation (16)).

(16) $$\sigma_{cmass}=Am_{b}^{B}$$

where both parameters *A* and *B* are undetermined coefficients, and can be determined by data fitting.

**Figure 4:**
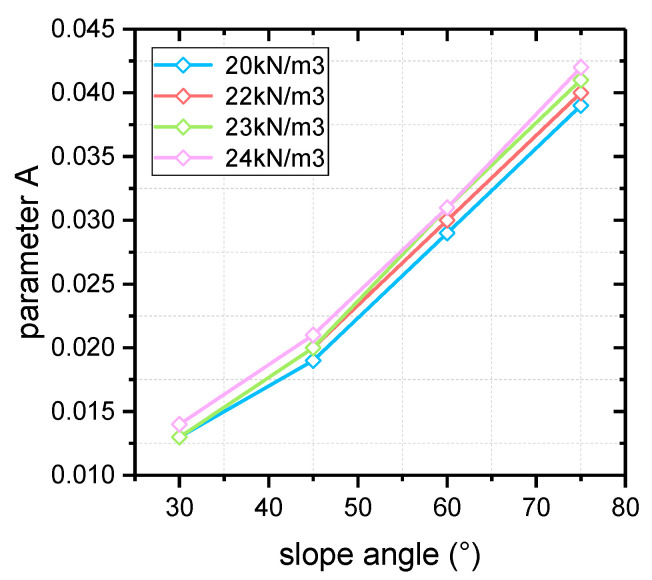
The variation in parameter *A* with slope angle.

**Figure 5:**
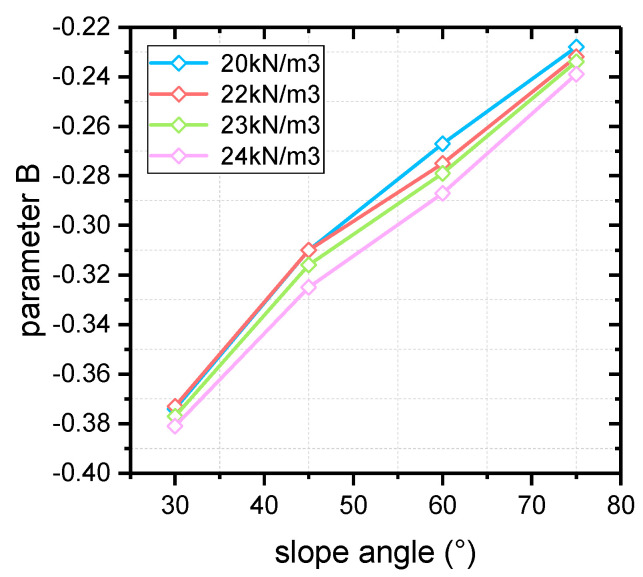
The variation in parameter *B* with slope angle.

The fitting results and parameter values of each curve in Figure 3 are shown in Table 3, and the variation laws of each parameter are shown in Figure 4 and Figure 5. From the value of *R*^2^ corresponding to each curve, the fitting curves show good matching with the critical data, which explains the reliability of the fitting results to a certain extent. In addition, it is obvious that the fitting values of slope critical curve parameters *A* and *B* are roughly the same under different slope unit weight, with slight changes. The slope angle has a stronger influence on the critical curve parameters, both of which increase with slope inclination. Also, it can be seen from Figure 4 and Figure 5 that the values of parameters *A* and *B* are approximately linearly positively correlated with the slope angle.

### 2.2. Nonlinear Reduction Method

As previously described, there are countless possibilities for a stable rock slope that causes the initial strength of rock mass to decay to critical strength. Without considering other external factors, the faster the strength of natural rock slope decays, the more likely it is to fail. Therefore, based on the position relationship between the initial strength of rock mass and the *σ_cmass_*-*m_b_* critical curve in the coordinate system, the corresponding reduction principle of parameters *σ_cmass_* and *m_b_* can be established by assuming that *σ_cmass_* and *m_b_* are reduced along the shortest path from the critical curve during slope strength attenuation. That is the shortest reduction path theory.

The distance from the initial state of slope to the critical strength curve can be calculated as

(17) $$D=\sqrt{{\left(m_{b}^{initial}-m_{b}^{critical}\right)}^{2}+{\left(\sigma_{cmass}^{initial}-\sigma_{cmass}^{critical}\right)}^{2}}$$

In the case that the initial *σ_cmass_* and *m_b_* are known, the critical values of *σ_cmass_* and *m_b_* can be obtained by substituting Equation (16) into Equation (17) and minimizing *D*. Then, the reduction ratio *ε* of *σ_cmass_* and *m_b_* can be solved by Equation (18).

(18) $$\varepsilon =\left(\frac{\sigma_{cmass}^{initial}}{\sigma_{cmass}^{critical}}\right)/\left(\frac{m_{b}^{initial}}{m_{b}^{critical}}\right)$$

From Figure 3, the slope strength critical curve is not unique, so the reduction ratio *ε* is not consistent either for different slopes. One of the cores of the nonlinear strength reduction method based on the Hoek–Brown criterion studied in this paper is just the determination of the reduction ratio *ε*. By ensuring that the reduction coefficients of *σ_cmass_* and *m_b_* meet the ratio relation *ε* at each reduction step, the numerical calculation of rock slope stability analysis conforms to the shortest path hypothesis.

As Equation (19) indicated, reducing *σ_cmass_* by coefficient *k_σ_* can be realized by reducing *σ_ci_*.

(19) $$\frac{\sigma_{cmass}}{k_{\sigma }}=\frac{\sigma_{ci}}{k_{\sigma }}\cdot s^{a}$$

While according to Equation (20), the reduction coefficient *k_mb_* of parameter *m_b_* in each iteration can be calculated (see Equation (22)).

(20) $$k_{m_{b}}=\frac{k_{\sigma }}{\varepsilon }$$

The reduction strategy of Equation (20) can be implemented in FLAC^3D^ by programming a custom rock slope strength reduction program in Fish language. Furthermore, it is inevitable to consider the method of determining the safety factor of slope because the parameter reduction is not synchronous. The classic definition in Equation (6) points out that the safety factor of slope *Fs* can be evaluated by the ratio of sliding resistance to sliding movement on the potential sliding surface, while the sliding resistance approximates to sliding movement at slope failure, which signifies that *Fs* is also be expressed by the ratio of initial sliding resistance *τ_initial_* to critical sliding resistance *τ_critical_* (see Equation (21)).

(21) $$Fs=\frac{\int_{l}\tau^{initial}dl}{\int_{l}\tau^{critical}dl}$$

The assessment of Equation (21) strongly relies on the positioning of the potential sliding surface, which can be located through various methods, such as displacement contour, maximum shear strain and maximum shear strain increment [73,74]. In this study, the horizontal displacement of the node of the slope model was adopted as the index to judge the position of slope potential sliding surface. Once the potential sliding surface is located, the coordinate information of each node on the sliding surface can be extracted, and the corresponding minor principal stress can be output. Then, the antisliding force on the potential sliding surface can be calculated by Equations (8) and (9), and the safety factor of slope can be solved by Equation (21). The specific steps are as follows: (1) traversing all grid nodes of the slope model after stability calculation to derive the coordinates and horizontal displacements of each node; (2) importing the position and displacement information of the derived nodes into MATLAB computing software, and processing by *k*-means clustering algorithm to find those nodes consisting of potential sliding surfaces; and (3) positioning the elements on the potential sliding surface to derive the corresponding minimum principal stress information, which is successively substituted into Equations (8), (9) and (21) to solve the slope safety factor.

## 3. Results and Discussion

**Figure 6:**
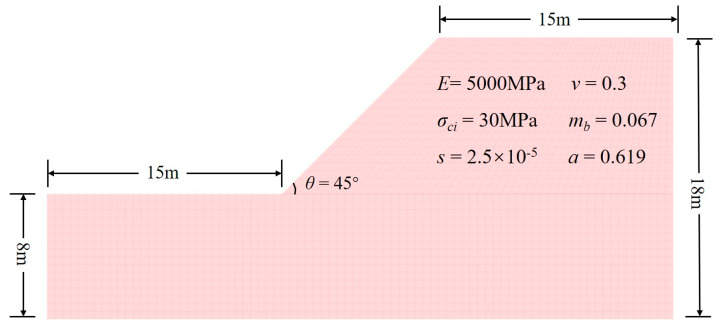
The simple slope model in Hoek–Brown material.

To verify the correctness of the Hoek–Brown criterion-based nonlinear reduction method proposed in this paper, it is necessary to employ the slope example whose safety factor has been confirmed for stability analysis. The built-in example in FLAC^3D^, simple slope in Hoek–Brown material, was selected and is shown in Figure 6. The uniformity of calculated safety factors of slope example by various methods in Table 4 explains that the calculation result by Hoek–Brown local linearization is relatively correct, which was therefore employed as the indicator to evaluate the efficacy of proposed method in this study.

**Figure 7:**
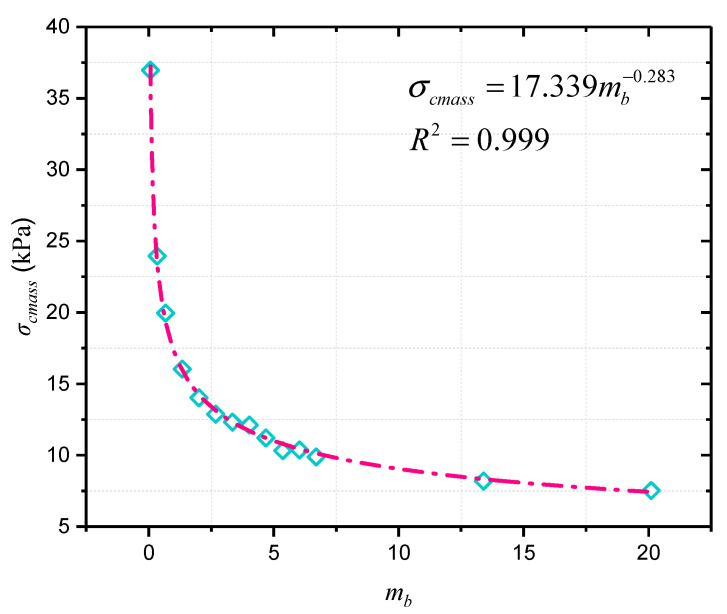
The critical points of *σ_cmass_* and *m_b_* for slope example.

The same slope model was established by FLAC^3D^, and corresponding constitutive model and parameters were assigned. The slope strength was adjusted to reach the critical state so as to obtain the critical strength curve and its expression of this slope example. There are two methods to determine the critical strength curve expression of slope. One is to repeat the above operations to obtain a large number of data points about the critical parameters *σ_cmass_* and *m_b_*, and to fit the critical strength curve expression of the model according to Equation (16). Secondly, the values of parameters *A* and *B* can also be theoretically obtained by solving the equations with only two sets of critical parameters *σ_cmass_* and *m_b_*, approximately determining the critical curve expression. The latter is simple and less time-consuming, but curve accuracy cannot be guaranteed. For the sake of precision, the first method was used to obtain multiple sets of *σ_cmass_* and *m_b_*, and the expression was fitted, as shown in Figure 7. Note that, the unit of *σ_cmass_* is set as kPa to avoid gaining values too small for the parameters *A* and *B* by fitting.

The initial state of slope example (*σ_cmass_*, *m_b_*) is (42.5056, 0.067). According to Equation (17), the distance from the initial state to the critical curve is

(22) $$D_{example}=\sqrt{{\left(0.067-m_{b}^{critical}\right)}^{2}+{\left(42.5056-17.339\times {\left(m_{b}^{critical}\right)}^{-0.283}\right)}^{2}}$$

**Figure 8:**
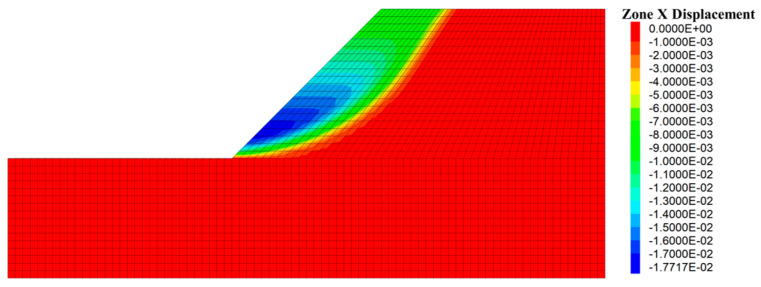
The stability calculation result and the horizontal displacement.

The calculation results show that during the range of *m_b_* (0, 25), the minimum *D_example_* approximates to 0.0267 when *m_b_^critical^* is 0.0421, and the corresponding value of *σ_cmass_^critical^* is 42.4964 kPa. By substituting the critical values into Equation (18), *ε* = 0.6285 were obtained, which indicates that when *σ_ci_* and *m_b_* are reduced in each iterative calculation of slope stability numerical analysis, the reduction ratio remains 0.6285. Afterwards, the FLAC^3D^ nonlinear reduction algorithm was customized by Fish language to solve the slope stability according to this reduction ratio, and the horizontal displacement is shown in Figure 8.

**Figure 9:**
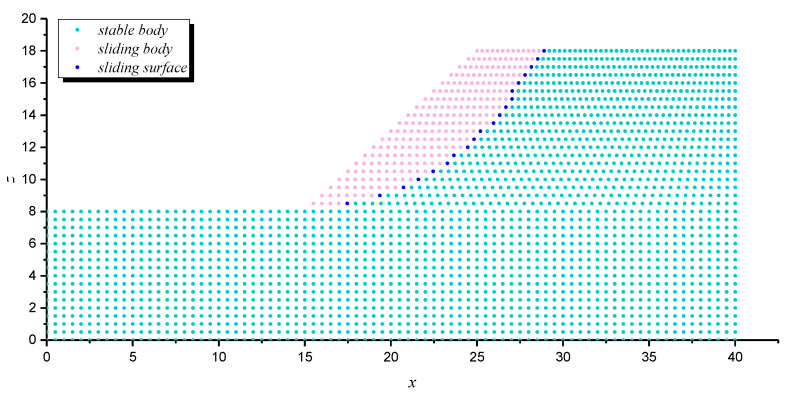
The clustering results and nodes on the potential sliding surface.

The coordinates and displacement data of each node in Figure 8 were derived and then imported into the MATLB calculation program. Then, the *k*-means clustering algorithm was used to classify these nodes according to the horizontal displacement differences, and corresponding nodes of sliding mass and stable mass were obtained, as shown in Figure 9. The nodes of sliding mass were isolated for analysis, and the rightmost nodes were considered the nodes on the potential sliding surface (dark blue nodes in Figure 9). The potential sliding surface of slope can be obtained by smoothing these nodes. According to the node coordinate information, the minimum principal stress of the node was derived from the FLAC^3D^ slope model, and the calculated safety factor of the slope example is 1.314. Compared with the safety factor calculated by other methods in Table 3, the error is about 14.26%, which proves the correctness of this method to some extent. The reason for the 14.26% error can be attributed to the fact that the potential sliding surface is determined by node displacement, while the safety factor must be solved according to the nearby stress-state elements. That is to say, the calculated safety factor of the slope example is susceptible to the grid density of the model. The shear–stress calculation of nodes may make the safety factor by this method more accurate; however, it seems to be difficult to realize in finite element software.

Similarly, the slope examples in [75] (Case 1) and [65] (Case 2) were selected to further demonstrate the feasibility and applicability of the proposed method. The slope parameters are presented in Table 5, and the same steps were implemented for these slope models, the results of which are presented in Table 6.

Meanwhile, except for Hoek–Brown local linearization reduction, the other Hoek–Brown nonlinear strength reduction methods, such as direct reduction of the involved Hoek–Brown parameters (*σ_ci_* and *m_b_*), equivalent Mohr–Coulomb parameter reduction was utilized to calculate the safety factor for proving the superiority of this Hoek–Brown-based strength nonlinear reduction method, and the calculated safety factors are presented in Table 7. Among these, the safety factors calculated by the proposed method are the closest to those by Hoek–Brown local linearization. The errors for slope cases are only 8.22% and 8.18%, respectively.

## 4. Conclusions

In response to the previous strength reduction methods on the Hoek–Brown criterion, which all use the same reduction factor for the parameters, this paper deduced a new reduction method that can reflect the nonlinear deterioration of the Hoek–Brown parameters during slope instability. The main findings of this paper are as follows.

(1) Critical strength curves for slopes show that uniaxial compressive strength of rock mass σcmass decreases with the increase in the Hoek–Brown parameter mb under different unit weight or slope angle conditions. On this basis, the general expression of slope critical strength curve was fitted by power function. Combined with the shortest reduction path theory, the ratio of the reduction coefficients of σcmass and mb can be determined.
(2) There are significant differences in the critical horizontal displacements between sliding and stable nodes of slope model. The k-means clustering algorithm was used to separate the sliding nodes and stable nodes according to such differences, so as to automatically identify the potential sliding surface. Then, the ratio of the sliding resistance to the sliding force was solved.
(3) Based on the proposed nonlinear reduction method and other known methods, stability calculations and safety factor comparisons were carried out on slope examples. Compared to other methods, the safety factors calculated by the proposed method differ less from the reference safety factor, which justifies the correctness and feasibility of this method to a certain extent.

## Figures and Tables

**Table 1:** Review of the previous reduction methods based on Hoek–Brown criterion.

Category	Description	Advantage	Disadvantage
Direct reduction method	Simultaneous reducing all or part of the Hoek–Brown parameters.	Simple and easy to use	Insufficient theoretical basis for parameter selection.
Strength envelope lowering	Overall reduction of the Hoek–Brown envelope by a factor.	Relatively accurate, and satisfying strength reduction concept	Inefficient calculations
Indirect reduction method	Reduction of equivalent Mohr–Coulomb parameters or instantaneous equivalent Mohr–Coulomb parameters.	Compatible with Mohr–Coulomb parameter-based criteria or software	Large errors in global equivalent parameters and complex calculation of instantaneous equivalent parameters

**Table 2:** Parameters of built-in slope in Hoek–Brown material.

E/(MPa)	*ν*	*m_b_*	*s*	*a*	*σ_ci_*/(MPa)
5000	0.3	0.067	0.000025	0.619	30

**Table 3:** Fitting results of slope critical curves.

Unit Weight/kN/m^3^	Slope Angle/°	*A*	*B*	*R* ^2^
20	30	0.013	−0.374	0.995
	45	0.019	−0.310	0.999
	60	0.029	−0.267	0.993
	75	0.039	−0.228	0.989
22	30	0.013	−0.373	0.996
	45	0.020	−0.310	0.999
	60	0.030	−0.275	0.996
	75	0.040	−0.232	0.984
24	30	0.013	−0.377	0.995
	45	0.020	−0.316	0.999
	60	0.031	−0.279	0.995
	75	0.041	−0.234	0.987
26	30	0.014	−0.381	0.995
	45	0.021	−0.325	0.999
	60	0.031	−0.287	0.997
	75	0.042	−0.239	0.988

**Table 4:** Factors of safety results for Hoek–Brown slope.

Resource	Method	Safety Factor
FLAC^3D^	Hoek–Brown local linearization	1.15
Hammah et al. [64]	Hoek–Brown envelop lowering	1.15
	Equivalent Mohr–Coulomb reduction	1.15
	Bishop simplified limit equilibrium	1.153
	Spencer limit equilibrium	1.152

**Table 5:** The parameters of other reported slope cases.

Slope Case	*H*/m	*θ*/°	*γ*/kN/m^3^	*E*/MPa	*ν*	*σ_ci_*/MPa	*m_b_*	*s*	*a*
Case 1 [75]	32	75	25	5000	0.3	40	0.281	2 × 10^−4^	0.508
Case 2 [65]	50	60	23	5000	0.3	10	0.657	4 × 10^−4^	0.522

**Table 6:** The calculation results of other slope cases.

Case	Expression of Critical Curve	Ratio of Reduction Coefficients	Potential Sliding Surface
1 [75]	$\sigma_{cmass}=0.303m_{b}^{-0.096}$	1.399	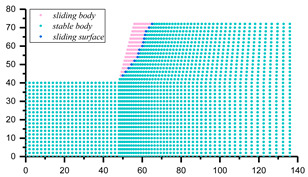
2 [65]	$\sigma_{cmass}=0.139m_{b}^{-0.213}$	1.104	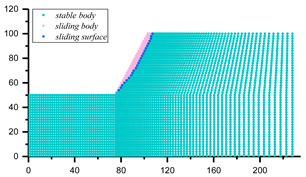

**Table 7:** The comparison between factor of safety by different methods.

Method	Factor of Safety Calculation			
	Hoek–Brown Local Linearization	Direct Reduction of *σ_ci_* and *m_b_*	Equivalent Mohr–Coulomb Reduction	Proposed Method
Case 1 [75]	1.5501	1.9659	1.9201	1.6775
Case 2 [65]	1.0090	0.9900	1.5070	1.0915

## Data Availability

The data used to support the findings of this study are available from the corresponding author upon request.
